# The Ring-LWE Problem in Lattice-Based Cryptography: The Case of Twisted Embeddings

**DOI:** 10.3390/e23091108

**Published:** 2021-08-26

**Authors:** Jheyne N. Ortiz, Robson R. de Araujo, Diego F. Aranha, Sueli I. R. Costa, Ricardo Dahab

**Affiliations:** 1 Institute of Computing, University of Campinas, Campinas 13083-852, Brazil; rdahab@ic.unicamp.br; 2 Federal Institute of São Paulo, Cubatão 11533-160, Brazil; robson.ricardo@ifsp.edu.br; 3 Department of Computer Science, Aarhus University, N 8200 Aarhus, Denmark; dfaranha@cs.au.dk; 4 Institute of Mathematics, Statistics and Computing Science, University of Campinas, Campinas 13083-859, Brazil; sueli@ime.unicamp.br

**Keywords:** lattice-based cryptography, twisted embeddings, ring learning with errors, spherical Gaussian sampling, ℤ*^n^*-equivalent lattices

## Abstract

Several works have characterized weak instances of the Ring-LWE problem by exploring vulnerabilities arising from the use of algebraic structures. Although these weak instances are not addressed by worst-case hardness theorems, enabling other ring instantiations enlarges the scope of possible applications and favors the diversification of security assumptions. In this work, we extend the Ring-LWE problem in lattice-based cryptography to include algebraic lattices, realized through twisted embeddings. We define the class of problems *Twisted Ring-LWE*, which replaces the canonical embedding by an extended form. By doing so, we allow the Ring-LWE problem to be used over maximal real subfields of cyclotomic number fields. We prove that Twisted Ring-LWE is secure by providing a security reduction from Ring-LWE to Twisted Ring-LWE in both search and decision forms. It is also shown that the twist factor does not affect the asymptotic approximation factors in the worst-case to average-case reductions. Thus, Twisted Ring-LWE maintains the consolidated hardness guarantee of Ring-LWE and increases the existing scope of algebraic lattices that can be considered for cryptographic applications. Additionally, we expand on the results of Ducas and Durmus (Public-Key Cryptography, 2012) on spherical Gaussian distributions to the proposed class of lattices under certain restrictions. As a result, sampling from a spherical Gaussian distribution can be done directly in the respective number field while maintaining its format and standard deviation when seen in $Z^{n}$ via twisted embeddings.

## 1. Introduction

Lattice-based cryptography comprehends the class of cryptosystems whose security is based on the conjectured intractability of hard lattice problems such as the Shortest Independent Vectors Problem (SIVP), the Shortest Vector Problem (SVP), and the Closest Vector Problem (CVP) [1,2]. The main computational problem in the foundation of most modern lattice-based cryptosystems is Learning with Errors (LWE) [3]. Since its introduction in the cryptographic realm in 2005, algebraically structured variants have been proposed, such as Learning with Errors over Rings [4], denoted Ring-LWE, and Module-LWE [5,6,7], among others [8].

Although the Ring-LWE hardness results hold for any number field [4,9], its most used instantiation in lattice-based cryptosystems is over power-of-two cyclotomic number fields, as evidenced by the finalists of NIST’s Post-Quantum Cryptography standardization effort [10]. This choice of a number field is particularly interesting because its ring of integers is isomorphic to the polynomial ring $R=Z\left[x\right]/\left(x^{n}+1\right)$, for *n* a power of two. The fact that $x^{n}+1$ is maximally sparse allows efficient polynomial multiplication using the number-theoretic transform combined with the negacyclic convolution. In addition to that, the transformation from the ring *R* to its dual, denoted $R^{\vee }$, is a simple scaling of the form $R=mR^{\vee }$, allowing applications to work directly on *R*, with no loss in their underlying worst-case hardness guarantees [4].

Another advantage of power-of-two cyclotomic number fields is that the sampling of error terms can be performed directly in the ring *R* considering a power basis, since the transformation to the associated vector subspace *H* isomorphic to $R^{n}$ is just a rigid rotation followed by scaling. For other choices of cyclotomic fields, sampling from a spherical Gaussian distribution can be done in an extended ring and performing a reduction modulo the cyclotomic polynomial $\Phi_{m}\left(x\right)$, which leads to the desired spherical distribution in the canonical embedding [11]. For general number fields, the best option in terms of security still is a sampling from an error distribution in *H* and computing the inverse transformation with respect to the canonical embedding [4,12].

There are several works in the literature exploring properties of number fields used in the foundation of some cryptosystems based on ideal lattices. An example is a quantum polynomial-time algorithm to find a small generator of a principal ideal in the ring of algebraic integers of cyclotomic rings [13], which applies to a few schemes including the fully-homomorphic encryption scheme of Smart and Vercauteren [14]. Moreover, a sequence of works has characterized weak instances of Ring-LWE and Poly-LWE problems and proposed attacks using special properties for specific parameters [15,16,17,18,19,20,21,22,23,24]. Another motivation for searching for alternative number fields is the inflexibility of system parameters that grow as a power-of-two. In such cryptosystems, when it is required to increase the security level, it may be necessary to increase the lattice dimension which implies doubling its size. However, a more suitable dimension could be a value much smaller than the next power of two. In fact, a ring dimension ranging from 700 to 800 suffices for 128-bit security [25].

Although these weak instances are not addressed by worst-case hardness theorems [26], new proposals adopting non-conventional rings have emerged as alternatives, thus favoring the diversification of security assumptions. For NTRU-based schemes, examples are the NTTRU [27], the third-round NTRU submission [28] in the NIST Post-Quantum Cryptography contest [10], and NTRU Prime [29]. For Ring-LWE, the instantiations have been restricted to cyclotomic number fields. Lyubashevsky, Peikert, and Regev introduced a toolkit with techniques for secure implementation of Ring-LWE primitives over any cyclotomic number field [12], allowing applications to work on cyclotomic rings with non-power-of-two dimension. Later on, this toolkit was implemented in software in two distinct libraries [30,31]. An alternative instantiation could be the adoption of the polynomial ring $Z\left[x\right]/\left(x^{p}-x-1\right)$ for *p* prime, which was proposed for NTRU Prime [29], and suggested for the Ring-LWE setting [32]. In this sense, we conjecture whether the Ring-LWE problem could be parameterized by number fields other than the cyclotomic for cryptographic applications.

### 1.1. Contributions

In this context, we extend the Ring-LWE class of problems to embrace more general algebraic constructions of lattices which allow additional factors on the embedding coordinates. We replace the canonical embedding by *twisted embeddings*. Since the canonical embedding is a special case of twisted embeddings, this replacement maintains the consolidated results for Ring-LWE. Twisted embeddings have been useful in coding theory, since they allow the construction of algebraic lattices with improved properties for Rayleigh fading channels, providing high density, maximum diversity, and great minimum product distance [33,34,35].

We extend the Ring-LWE problem by replacing the canonical embedding with twisted embeddings on both the search and decision variants. As a result, we obtain the *Twisted Ring-LWE* problem, in which the error terms are sampled in the space *H* isomorphic to $R^{n}$ under the inner product induced by a twisted embedding. We show that Twisted Ring-LWE is at least as secure as Ring-LWE through a security reduction from Ring-LWE to Twisted Ring-LWE. We also recomputed the approximation factors in the worst-case to average-case reductions from hard lattice problems taking into account the new twist factor.

As a result, algebraic constructions from coding theory via twisted embeddings can also be used in cryptographic applications based on the Ring-LWE problem. In this work, we focused our attention on the algebraic construction of rotated $Z^{n}$-lattices via twisted embeddings. Ducas and Durmus [11] showed that a spherical Gaussian distribution in the ring $Q\left[x\right]/(\Theta_{m}\left(x\right))$, where $\Theta_{m}\left(x\right)=x^{m}-1$ if *m* is odd, and $\Theta_{m}\left(x\right)=x^{\frac{m}{2}}+1$ if *m* is even, corresponds to a distribution with the same format in the space *H*, but linearly wider in the ring dimension. This occurs because the lattice obtained from the ring $Q\left[x\right]/(\Theta_{m}\left(x\right))$ is a rotated $Z^{n}$-lattice in the canonical embedding. The same holds for the ring of integers of a power-of-two cyclotomic number field. Thus, we generalize this result of Ducas and Durmus by showing that if the parameter ring leads to a rotated $Z^{n}$-lattice under twisted embeddings, then both the format and the standard deviation of a spherical Gaussian distribution in $K_{R}$ is preserved when seen in *H*. Examples of ideal lattices equivalent to $Z^{n}$ are those obtained from power-of-two cyclotomic number fields [36], and their maximal real subfields [37], and the maximal real subfields of *p*-th cyclotomic number fields. Since power-of-two cyclotomic rings have been widely used in cryptographic applications, we consider parameterizing the Ring-LWE problem with the ring of integers of the maximal real subfield of a cyclotomic number field. We discuss the limitations of using maximal real subfields in a public-key encryption scheme [12] using the polynomial representation in terms of the arithmetic operations and the expansion factor of the defining polynomial. However, we argue that these limitations could be circumvented by using the coefficient vector representation, as done in [12]. Finally, we also argue that twisted embeddings can be used as a tool to connect Ring-LWE instances over distinct rings, which may lead to a response to the open question left by Peikert, Regev, and Stephens-Davidowitz [9]. In fact, if the parameter rings generate the same algebraic lattice in the space *H*, their Ring-LWE instances can be efficiently converted between themselves.

### 1.2. Organization

This paper is organized as follows. Section 2 is devoted to the introduction of concepts and results on lattices and algebraic number theory to be used throughout the paper. In particular, Section 2.4 presents the original statement of the Ring-LWE problem in its search and decision variants, and also the computational problems which form the foundation of the (Ring)-LWE hardness.

Section 3 introduces the twisted embeddings and generalizes the class of Ring-LWE problems by adopting twisted embeddings. We prove that multiplying the coordinates of vectors in the canonical representation by a twisting factor does not affect the hardness of Ring-LWE. This is shown via a reduction from both search and decision versions of Ring-LWE to their corresponding twisted forms. Moreover, we compute the new approximation factors for the reduction from SIVP to DGS (Discrete Gaussian Sampling problem), and also for the reduction from DGS to Ring-LWE. Since the new approximation factors are simply multiplied by a scalar associated with the lattice dimension *n*, the asymptotic factors are not affected by the change of embeddings.

Section 4 extends to a more general class of number fields the results of Ducas and Durmus on spherical Gaussian sampling [11]. We show that correct noise sampling can be performed directly in the field representation of lattices equivalent to $Z^{n}$ without any increase in the standard deviation. Section 4.1 discusses the practical impacts of instantiating the Ring-LWE problem over the ring of integers of the maximal real cyclotomic number field $Q(\zeta_{p}+\zeta_{p}^{-1})$, where p≥5 is a prime number. We analyze the main computational operations in the compact public-key cryptosystem of Lyubashevsky, Peikert, and Regev [12], and also the format of the ring’s defining polynomial in terms of the expansion factor. Finally, Section 5 discuss our results and highlight future research directions on the practical aspects of the Twisted Ring-LWE problem.

## 2. Preliminaries on Lattices and Algebraic Number Theory

In this section, we introduce concepts, results and notation to be used throughout the paper. For a positive integer number *m*, denote by [m] the set {1,2,…,m}. For 1≤p<∞, the $\ell_{p}$-norm of a vector a in $R^{n}$ or $C^{n}$ is ${∥a∥}_{p}={\left(\sum_{i=1}^{n}{\left|a_{i}\right|}^{p}\right)}^{1/p}$, and the $\ell_{\infty }$-norm is ${∥a∥}_{\infty }=\max_{i\in [n]}\left|a_{i}\right|$.

### 2.1. The Space H

Frequently, lattices are defined in the Euclidean space $R^{n}$. However, in the Ring-LWE context [4,9], it is more convenient to define lattices in a specific subspace of $C^{n}$ isometric to $R^{n}$: the *space H*.

**Definition** **1** (Space H)**.** *Let $s_{1}$ and $s_{2}$ be non-negative integer numbers such that $n=s_{1}+2s_{2}>0$. The *subspace $H\subseteq C^{n}$*is defined as*

$$H=\left\{\left(a_{1},a_{2},\ldots ,a_{n}\right)\in R^{s_{1}}\times C^{2s_{2}}:a_{j+s_{1}+s_{2}}=\bar{a_{j+s_{1}}},\;\forall \;j\in \left[s_{2}\right]\right\}.$$

We consider *H* endowed with the inner product obtained as a restriction of the standard inner product of $C^{n}$:

$${\langle a,b\rangle }_{H}:=\sum_{i\in [n]}a_{i}\bar{b_{i}}=\sum_{i\in [s_{1}]}a_{i}b_{i}+\sum_{j\in [s_{2}]}\left(a_{j+s_{1}}b_{j+s_{1}+s_{2}}+a_{j+s_{1}+s_{2}}b_{j+s_{1}}\right)\in R.$$

The norm (usually $\ell_{2}$-norm) of $a=(a_{1},a_{2},\ldots ,a_{n})\in H$ is defined as $∥a∥=\sqrt{{\langle a,a\rangle }_{H}}$.

For i∈[n], denote by $u_{i}$ the vector with all zero coordinates except for the *i*-th position, which is equal to one. We consider $\left\{u_{1},u_{2},\ldots ,u_{n}\right\}$ the canonical basis of $R^{n}$ (over R) and $C^{n}$ (over C). An orthonormal basis for *H* can be defined in terms of the canonical basis of $C^{n}$:

**Definition** **2** (Canonical basis of *H*)**.** *Let $s_{1}$ and $s_{2}$ be non-negative integer numbers such that $n=s_{1}+2s_{2}>0$. For $i\in [s_{1}]$, define $h_{i}=u_{i}$. For $i\in [s_{2}]$, define $h_{i+s_{1}}=\frac{1}{\sqrt{2}}\left(u_{i+s_{1}}+u_{i+s_{1}+s_{2}}\right)$ and $h_{i+s_{1}+s_{2}}=\frac{i}{\sqrt{2}}\left(u_{i+s_{1}}-u_{i+s_{1}+s_{2}}\right)$. Then, the set $B=\{h_{1},h_{2},\ldots ,h_{n}\}$ is an orthonormal basis of H, which we call the* canonical basis *of H as an n-dimensional R-vector space.*

Notice that any vector $a=\left(a_{1},a_{2},\ldots ,a_{n}\right)\in H\subseteq C^{n}$ can be written as an R-linear combination of the vectors of the canonical basis B of *H* as

$$a=\sum_{i\in [s_{1}]}a_{i}h_{i}+\sum_{i\in [s_{2}]}\sqrt{2}ℜ\left(a_{i+s_{1}}\right)h_{i+s_{1}}+\sum_{i\in [s_{2}]}\sqrt{2}ℑ\left(a_{i+s_{1}}\right)h_{i+s_{1}+s_{2}},$$

where ℜ(·) and ℑ(·) denote the real and imaginary parts of a complex number, respectively.

The linear map $\kappa \left(\sum_{i\in [n]}b_{i}h_{i}\right):=\sum_{i\in [n]}b_{i}u_{i}$, with $b_{i}\in R$, defines an isomorphism between the R-vector spaces *H* and $R^{n}$, such that ${\langle a,b\rangle }_{H}=\langle \kappa \left(a\right),\kappa \left(b\right)\rangle$, where 〈·,·〉 denotes the standard inner product in $R^{n}$. Then, it follows that *H* and $R^{n}$ are isometric, that is, *H* is an Euclidean space, as defined next. In particular, the norm of an element a∈H coincides with the usual norm ($\ell_{2}$-norm) of $\kappa \left(a\right)\in R^{n}$, that is, $∥a∥={∥\kappa \left(a\right)∥}_{2}$.

### 2.2. Lattices in Euclidean Vector Spaces

An *Euclidean vector space* $\left(E,{\langle \cdot ,\cdot \rangle }_{E}\right)$ is an *n*-dimensional R-vector space *E* with an inner product ${\langle \cdot ,\cdot \rangle }_{E}$, which is isometric to $R^{n}$ with the standard inner product. Consider an orthonormal basis $B\left(E\right)=\left\{e_{1},e_{2},\ldots ,e_{n}\right\}$ of *E*.

A set Λ⊂E is said to be a *full-rank lattice* (or simply *lattice*), if Λ is a discrete additive subgroup of *E* with rank *n*. Equivalently, Λ⊂E is a lattice if there exists a set of linearly independent vectors $B=\{v_{1},v_{2},\ldots ,v_{n}\}\subset E$ such that

$$\Lambda =\Lambda \left(B\right)=\left\{\sum_{i\in [n]}a_{i}v_{i}\;:\;a_{i}\in Z\right\}.$$

The set B is called a *basis* (or a Z-basis) of Λ. For each $v_{j}\in B$, it can be written in terms of the orthonormal basis B(E) as $v_{j}=\sum_{i\in [n]}v_{ij}e_{i}$ for $v_{ij}\in R$.

The *minimum distance* of a lattice Λ in the $\ell_{p}$-norm, denoted $\lambda_{1}^{\left(p\right)}\left(\Lambda \right)$, is the length of a shortest nonzero lattice vector, that is, $\lambda_{1}^{\left(p\right)}\left(\Lambda \right)=\min_{0\neq x\in \Lambda }{∥x∥}_{p}$. Similarly, for any k≤n, the *k*-th *successive minimum* of a lattice Λ, denoted $\lambda_{k}^{\left(p\right)}\left(\Lambda \right)$, is the smallest $\hat{r}>0$ such that Λ contains at least *k* linearly independent vectors of norm at most $\hat{r}$.

The matrix $M={\left[v_{ij}\right]}_{n\times n}$, for which the *j*-th column is given by the coefficients of $v_{j}$ written in the orthonormal basis B(E), is called a *generator matrix* of Λ. Two basis generate the same lattice if and only if the associated generator matrices M and $M^{\prime }$ are related as $M^{\prime }=MU$, where U is unimodular (has integer entries and det(U)=±1). The matrix $G=M^{t}M$ is called the *Gram matrix* of Λ with respect to M. Since the basis B(E) of the Euclidean vector space is orthonormal, then $G={\left[{\langle v_{i},v_{j}\rangle }_{E}\right]}_{n\times n}$. The determinant of G is called the *determinant* of Λ and is denoted by det(Λ). Clearly, $\det\left(\Lambda \right)=\det{\left(M\right)}^{2}$ does not depend of a particular basis of Λ.

The *dual lattice* of Λ is the lattice $\Lambda^{*}=\left\{a\in E\;:\;{\langle a,b\rangle }_{E}\in Z,\forall \;b\in \Lambda \right\}$ and has generator matrix ${\left(M^{t}\right)}^{-1}$. It is known that ${\left(\Lambda^{*}\right)}^{*}=\Lambda$ and if Λ has generator matrix M, then ${\left(M^{t}\right)}^{-1}$ is a generator matrix for $\Lambda^{*}$ and therefore $\det\left(\Lambda^{*}\right)=\det{\left(\Lambda \right)}^{-1}$.

A lattice Λ⊂E is called *integral* if ${\langle a,b\rangle }_{E}\in Z$ for all a,b∈Λ. Equivalently, Λ is an integral lattice if and only if $\Lambda \subset \Lambda^{*}\subset \left(\Lambda /\det(\Lambda )\right)$. An integral lattice is called *unimodular*, or *self-dual*, if det(Λ)=1 or, equivalently, if $\Lambda =\Lambda^{*}$.

Two lattices Λ and $\Lambda^{\prime }$ are said to be *equivalent* if one can be obtained from the other through a rotation, a reflection, or a change of scale. We denote this equivalence by $\Lambda ≃\Lambda^{\prime }$. Two Gram matrices G and $G^{\prime }$ of two equivalent lattices Λ and $\Lambda^{\prime }$, respectively, are related as $G^{\prime }=c^{2}U^{t}GU$, where c≠0 is a real constant and U is unimodular.

We say that a lattice Λ in $\left(E,{\langle \cdot ,\cdot \rangle }_{E}\right)$ is *orthogonal* if it has a basis $B=\{v_{1},v_{2},\ldots ,v_{n}\}$ such that $\langle v_{i},v_{j}\rangle =0$ if i≠j, for all i,j∈[n]. This means that Λ has a diagonal Gram matrix. Moreover, if the basis B satisfies $\langle v_{i},v_{j}\rangle =0$ if i≠j and $\langle v_{i},v_{j}\rangle =c$ if i=j, for all i,j∈[n] and c∈R, then Λ is equivalent to the $Z^{n}$-lattice. In this case, Λ has a Gram matrix $G=c{\mathrm{Id}}_{n}$. In particular, when c=1, we say that Λ is an *orthonormal* lattice.

#### Gaussian Measures

For r>0, define the Gaussian function $\rho_{r,c}:H\to \left(0,1\right]$ centered at c as

(1) $$\rho_{r,c}\left(a\right)={\exp(-\pi ∥a-c∥}^{2}/r^{2}).$$

The subscript c is taken to be 0 when omitted. By normalizing this function, we obtain the continuous Gaussian probability distribution $D_{r}$ of width *r*, whose density is given by $r^{-n}\cdot \rho_{r}\left(x\right)$.

We extend this definition to elliptical Gaussian distributions in ${\left\{h_{i}\right\}}_{i\in [n]}$ (the canonical basis of *H*) as follows. Let $r=\left(r_{1},\ldots ,r_{n}\right)\in {\left(R^{+}\right)}^{n}$ be a vector of positive real numbers such that $r_{j+s_{1}+s_{2}}=r_{j+s_{1}}$ for each $j\in [s_{2}]$. Then, a sample from the *n*-dimensional distribution $D_{r}$ is given by $\sum_{i\in [n]}x_{i}h_{i}$, where the $x_{i}$ are chosen independently from the (one-dimensional) Gaussian distribution $D_{r_{i}}$ over R.

The smoothing parameter is a lattice parameter defining the width beyond which a discrete Gaussian starts to behave similarly to a continuous distribution [38]. It is related to the minimum distance and the successive minimum of a lattice and it will be used to derive the approximation factors in the worst-case to average-case reduction for to the Twisted Ring-LWE problem. The Gaussian mass of a coset c+Λ is defined as $\rho_{r}\left(c+\Lambda \right)=\sum_{x\in c+\Lambda }\rho_{r}\left(x\right)$.

**Definition** **3** (Smoothing parameter)**.** *For an n-dimensional lattice* *Λ* *and positive real ϵ>0, the* smoothing parameter $\eta_{\epsilon }\left(\Lambda \right)$ is the smallest r such that $\rho_{1/r}\left(\Lambda^{*}\backslash \left\{0\right\}\right)\leq \epsilon$.

For any $c\in R^{n}$, real r>0, and an arbitrary lattice Λ with dimension *n*, normalizing the Gaussian function $\rho_{r,c}\left(a\right)$ gives the *discrete Gaussian distribution* over Λ as

$$D_{\Lambda ,r,c}\left(a\right)=\frac{\rho_{r,c}\left(a\right)}{\rho_{r,c}\left(\Lambda \right)},$$

for all a∈Λ.

### 2.3. Algebraic Number Theory

In this section, we summarize concepts and results from algebraic number theory, presenting as an example the case of cyclotomic number fields and their maximal real subfields. Details can be found in [39,40].

An (*algebraic*) *number field* *K* is a finite extension of the field Q. This means that Q⊂K and *K* is a Q-vector space with finite dimension. The *degree* of *K*, denoted [K:Q], is the dimension of the Q-vector space *K*. In general, if *K* and *L* are number fields such that K⊂L, the symbol [L:K] is defined to be the integer number [L:Q]/[K:Q] and is called the degree of the extension L/K.

By the Primitive Element Theorem, there exists an element θ∈K such that K=Q(θ), which is equivalent to say that $\left\{1,\theta ,\theta^{2},\ldots ,\theta^{n-1}\right\}$, with n=[K:Q], is a *power basis* of *K* over Q. Also, if p(x) is the minimal polynomial of θ over Q, then *K* is isomorphic to Q[x]/(p(x)) and $K=Q(\theta^{\prime })$ for some root $\theta^{\prime }$ of p(x). The roots of p(x) are called the conjugates of θ.

**Example** **1** (Cyclotomic number field)**.** *A number field of particular interest is $Q(\zeta_{m})$, the* *m*-th cyclotomic field*, where $\zeta_{m}=\exp\left(2\pi i/m\right)$ is a primitive m-th root of unity for any integer number m≥1. The degree of $Q(\zeta_{m})$ is φ(m), where φ(·) denotes Euler’s totient function. The minimal polynomial of $\zeta_{m}$, called the* *m*-th cyclotomic polynomial, is $\Phi_{m}\left(x\right)=\prod_{k\in Z_{m}^{*}}\left(x-\zeta_{m}^{k}\right)$, where $Z_{m}^{*}$ denotes the group of invertible elements in $Z_{m}$.

**Example** **2** (Maximal real subfield)**.** *For m≢2(mod4), m>1, the number field $Q\left(\zeta_{m}+\zeta_{m}^{-1}\right)\subset R\cap Q\left(\zeta_{m}\right)$ is the* maximal real subfield *of $Q(\zeta_{m})$ and has degree φ(m)/2.*

Let *K* be a number field. A map $\;\bar{}:K\to K$ is called an *involution* of *K* if $\bar{a+b}=\bar{a}+\bar{b}$, $\bar{a\cdot b}=\bar{a}\cdot \bar{b}$, and $\bar{\bar{a}}=a$, for all a,b∈K. If K=C, the complex conjugation is an example of involution. If $K=Q(\zeta_{m})$ is a cyclotomic number field, then $\bar{\zeta_{m}}=\zeta_{m}^{-1}$ is the same involution given by the complex conjugation. In this work, whenever the cyclotomic number field is used, we implicitly assume this involution. For the maximal real subfield $Q(\zeta_{m}+\zeta_{m}^{-1})$, we consider the involution given by the identity map.

The subfield $F=\{a\in K|\bar{a}=a\}$, called the fixed field by involution of *K*, satisfies [K:F]≤2. When [K:F]=1 (or F=K), we say that the involution is *trivial* (it is the identity); otherwise, the involution is said to be *non-trivial*. If $K=Q(\zeta_{m})$, the fixed field by the involution $\bar{\zeta_{m}}=\zeta_{m}^{-1}$ of *K* is its maximal real subfield [36].

#### 2.3.1. Field Monomorphisms

Let *K* be a number field of degree *n*. There are exactly *n* distinct monomorphisms (of fields) from *K* to C. These monomorphisms are Q-monomorphisms. If K=Q(θ) and p(x) is the minimal polynomial of θ, these monomorphisms can be defined as $\sigma_{i}\left(\theta \right)=\theta_{i}$ for i∈[n], where $\theta_{i}$ are all the distinct roots of p(x).

A monomorphism $\sigma_{i}:K\to C$ is said to be *real* if $\sigma_{i}\left(K\right)\subset R$. Otherwise, it is said to be *complex*. If $\sigma_{i}$ is a complex monomorphism, then $\bar{\sigma_{i}}$ is another complex monomorphism defined by $\bar{\sigma_{i}}\left(a\right)=\bar{\sigma_{i}\left(a\right)}$. So, we can write the degree *n* as $n=s_{1}+2s_{2}$, where $s_{1}\geq 0$ is the number of real monomorphisms and $2s_{2}\geq 0$ is the number of complex monomorphisms from *K* to C. The *canonical embedding* from *K* into the subspace *H* is the homomorphism

$$\sigma \left(a\right)=\left(\sigma_{1}\left(a\right),\sigma_{2}\left(a\right),\ldots ,\sigma_{n}\left(a\right)\right).$$

Its image is a lattice, used in the Ring-LWE problem [4,9].

The pair $\left(s_{1},s_{2}\right)$ is called the *signature* of *K*. We say that *K* is *totally real* when $s_{2}=0$, and that *K* is *totally complex* when $s_{1}=0$. The number field *K* is said to be a *CM-field* if it is totally complex and has degree two over its fixed field by the involution *F* [36].

Any cyclotomic number field $K=Q(\zeta_{m})$, with m≥3, is totally complex. Their monomorphisms are defined as $\sigma_{i}\left(\zeta_{m}\right)=\zeta_{m}^{i}$ for each i∈[m] such that gcd(i,m)=1. In turn, any maximal real cyclotomic subfield $Q(\zeta_{m}+\zeta_{m}^{-1})$ is totally real. Their monomorphisms are defined as $\sigma_{i}\left(\zeta_{m}+\zeta_{m}^{-1}\right)=\zeta_{m}^{i}+\zeta_{m}^{-i}$ for each $i\in \left[\left\lfloor m/2\right\rfloor \right]$ such that gcd(i,m)=1. Note that $Q(\zeta_{m})$ is a CM-field once $Q(\zeta_{m})$ is a totally complex field of degree two over $Q(\zeta_{m}+\zeta_{m}^{-1})$.

The number field *K* is said to be a *Galois* number field if, for every x∈K, the minimal polynomial of *x* over Q has all its roots in *K*. In this case, the set of automorphisms σ:K→K, where σ(a)=a for all a∈Q, constitutes a group under the composition, called *Galois group* of *K* over Q and denoted by Gal(K/Q). If K⊂C is a Galois number field, then the monomorphisms from *K* to C are exactly the elements of Gal(K/Q). An important fact is that any Galois number field is totally real or totally complex. Cyclotomic number fields and their maximal real subfields are Galois number fields. Specifically, the set $\mathrm{Gal}(Q\left(\zeta_{m}\right)/Q)$ is isomorphic to $Z_{m}^{*}$ and $\mathrm{Gal}(Q\left(\zeta_{m}+\zeta_{m}^{-1}\right)/Q)$ is isomorphic to $Z_{m}^{*}/\left\{\pm 1\right\}$.

#### 2.3.2. Ring of Integers and Its Ideals

Let *K* be a Galois number field. For every a∈K, the *trace* and *norm* of any element a∈K can be defined, respectively, as

$${\mathrm{Tr}}_{K}\left(a\right)=\sum_{\sigma \in \mathrm{Gal}(K/Q)}\sigma \left(a\right)\;\mathrm{and}\;N_{K}\left(a\right)=\prod_{\sigma \in \mathrm{Gal}(K/Q)}\sigma \left(a\right).$$

For all a∈K, ${\mathrm{Tr}}_{K}\left(a\right)$ and $N_{K}\left(a\right)$ are elements of Q.

The set of all elements in a number field *K* that are the root of a monic polynomial in Z[x] is a ring called the *ring of integers* of *K*, denoted by $O_{K}$. If *K* is a number field of degree *n*, its ring of integers has a Z-basis with *n* elements, which is called an *integral basis* of *K*. If $a\in O_{K}$, then ${\mathrm{Tr}}_{K}\left(a\right)$ and $N_{K}\left(a\right)$ are elements of Z.

If I is a nonzero (integral) ideal of $O_{K}$, then I has a Z-basis with *n* elements. The same holds if I is a *fractional ideal* of *K*, which is a subset of *K* satisfying the condition that $dI\subset O_{K}$ is an integral ideal for some element $d\in O_{K}$. Note that every integral ideal is also fractional (d=1). Also, any Z-basis of some nonzero fractional ideal of *K*, including its ring of integers, is a Q-basis of *K*. If $K=Q(\zeta_{m})$ is the *m*-th cyclotomic number field, then $O_{K}=Z\left[\zeta_{m}\right]$, which is the set of all Z-linear combinations of powers of $\zeta_{m}$. Similarly, the ring of integers of $Q(\zeta_{m}+\zeta_{m}^{-1})$ is $Z[\zeta_{m}+\zeta_{m}^{-1}]$. In general, the ring of integers of a number field K=Q(θ) does not have the form Z[θ]. When this is the case, we say that *K* is a *monogenic* number field.

The fractional ideal $D_{K}^{-1}=\left\{a\in K:{\mathrm{Tr}}_{K}\left(aO_{K}\right)\subset Z\right\}$ is the *codifferent ideal*, that is, the dual ideal of the ring of integers. Frequently, the codifferent ideal is also denoted by $O_{K}^{\vee }$. Note that $O_{K}\subset D_{K}^{-1}$. If $O_{K}=Z\left[\theta \right]$ for some θ∈K, then $O_{K}^{\vee }={\left(p^{\prime }\left(\theta \right)\right)}^{-1}O_{K}$, where $p^{\prime }\left(x\right)$ is the derivative of the minimal polynomial p(x) of θ [41] (Section 13.2, J). The inverse ideal of the codifferent, that is, $D_{K}={\left(D_{K}^{-1}\right)}^{-1}$, is an ideal of $O_{K}$ called *different* of *K*. In general, the *dual ideal* of any fractional ideal I of *K* is the fractional ideal $I^{\vee }$ of *K*, defined as

$$I^{\vee }:=\left\{a\in K:{\mathrm{Tr}}_{K}\left(aI\right)\subset Z\right\}=I^{-1}\cdot O_{K}^{\vee }.$$

If I is a nonzero fractional ideal of $O_{K}$, the norm of I is $N\left(I\right)=|O_{K}/I|$ (the cardinality of the quotient of additive groups). If I and J are ideals of $O_{K}$, then N(IJ)=N(I)N(J), where IJ denotes the product of IandJ, that is, the set all finite sums of products *ab* for a∈I and b∈J. If I is a principal ideal generated by some a∈K, then $N\left(I\right)=|N_{K}\left(a\right)|$.

### 2.4. The Ring-LWE Problem

In the following definitions, a lattice Λ is usually represented by a basis B and, in the context of algebraic lattices, Λ can be seen as a fractional ideal I of an arbitrary number field *K* via canonical embedding.

Firstly, we define the computational problems which form the foundation of the (Ring)-LWE hardness, namely the decision version of the Shortest Vector Problem (GapSVP), the Shortest Independent Vectors Problem (SIVP), and the Discrete Gaussian Sampling (DGS) problem, which is denoted *K*-DGS when the underlying lattice is taken over a number field *K* [4].

**Definition** **4** (GapSVP${}_{\gamma }$)**.** *For an approximation factor γ=γ(n)≥1, the GapSVP${}_{\gamma }$ is: given a lattice *Λ* and length d>0, output YES if $\lambda_{1}\left(\Lambda \right)\leq d$ and NO if $\lambda_{1}\left(\Lambda \right)>\gamma d$.*

**Definition** **5** (SIVP${}_{\gamma }$)**.** *For an approximation factor γ=γ(n)≥1, the SIVP${}_{\gamma }$ is: given a lattice *Λ*, output n linearly independent lattice vectors of length at most $\gamma \left(n\right)\cdot \lambda_{n}\left(\Lambda \right)$.*

By seeing a fractional ideal I of an arbitrary number field *K* as a lattice using the canonical embedding, let $D_{I,r}$ denote the discrete Gaussian distribution of width *r* over I in the field tensor product $K_{R}=K{⊗}_{Q}R$, which is isomorphic to the space *H*.

**Definition** **6** (*K*-DGS${}_{\gamma }$)**.** *For a function γ that maps lattices to nonnegative reals, the K-DGS${}_{\gamma }$ problem is: given an ideal I in K and a parameter r≥γ=γ(I), output an independent sample from a distribution that is within negligible distance of $D_{I,r}$.*

Alternatively, for the purpose of the worst-case to average-case reduction for (Ring-)LWE, the DGS problem can be stated as follows: given an *n*-dimensional lattice Λ and a number $r\geq \sqrt{2n}\cdot \eta_{\epsilon }\left(\Lambda \right)/\alpha$, output a sample from $D_{\Lambda ,r}$.

In order to define the Ring-LWE distribution and the computational problems associated with it, let *K* be a number field with ring of integers $R=O_{K}$. Recall that $R^{\vee }$ is the (fractional) codifferent ideal of *K*, and let $T=K_{R}/R^{\vee }$. Let q≥2 be a (rational) integer modulus and, for any fractional ideal I of *K*, let $I_{q}=I/qI$.

**Definition** **7** ([4] Ring-LWE distribution)**.** *For $s\in R_{q}^{\vee }$ (the “secret”) and an error distribution ψ over $K_{R}$, a sample from the Ring-LWE distribution $A_{s,\psi }$ over $R_{q}\times T$ is generated by choosing $a\leftarrow R_{q}$ uniformly at random, choosing e←ψ, and outputting $\left(a,b=\left(a\cdot s\right)/q+e\;\mathrm{mod}\;R^{\vee }\right)$.*

**Definition** **8** ([4] Ring-LWE, search)**.** *Let *Ψ* be a family of distributions over $K_{R}$. The search version of the Ring-LWE problem, denoted R-LWE${}_{q,\Psi }$, is defined as follows: given access to arbitrarily many independent samples from $A_{s,\psi }$, for some arbitrary $s\in R_{q}^{\vee }$ and ψ∈Ψ, find s.*

**Definition** **9** ([4,9] Ring-LWE, average-case decision)**.** *Let *Υ* be a distribution over a family of error distributions, each over $K_{R}$. The average-case Ring-LWE decision problem, denoted R-LWE${}_{q,\Upsilon }$, is to distinguish (with non-negligible advantage) between independent samples from $A_{s,\psi }$ for a random choice of $\left(s,\psi \right)\leftarrow U\left(R_{q}^{\vee }\right)\times \Upsilon$, and the same number of uniformly random and independent samples from $R_{q}\times T$.*

## 3. The Twisted Ring-LWE

Firstly, we collect important results on algebraic lattices obtained through twisted embeddings. Then, we present the class of problems Twisted Ring-LWE, which is the main contribution of this work. The hardness of Twisted Ring-LWE is demonstrated by security reductions from the original Ring-LWE problem. Also, we recompute the approximation factors in the worst-case to average reduction from the SIVP problem, considering the twist factor defining the twisted embedding.

### 3.1. Twisted Embeddings

In this section consider the following setting. Let *K* be an algebraic number field with degree *n*, signature $\left(s_{1},s_{2}\right)$, and $\;\bar{}\;$ a fixed involution. Consider *F* to be the fixed field by the involution of *K*. Let $\sigma_{i}$ be the real monomorphisms for $i\in [s_{1}]$, and $\sigma_{i+s_{1}}$ be the complex monomorphisms for $i\in [2s_{2}]$ from *K* to C, where $\sigma_{i+s_{1}+s_{2}}=\bar{\sigma_{i+s_{1}}}$ for all $i\in [s_{2}]$. The twisted embeddings defined next are a generalization of the canonical embedding [36]. An element τ∈K is said to be *totally positive* if τ∈F and $\tau_{i}=\sigma_{i}\left(\tau \right)$ is a positive real number for all i∈[n].

**Definition** **10** (Twisted embeddings)**.** *For any totally positive τ∈F, the τ-*twisted embedding* (or simply* twisted embedding*) is the homomorphism $\sigma_{\tau }:K\to H$, defined as*

$$\begin{array}{cc} \sigma_{\tau }\left(a\right)= & (\sqrt{\tau_{1}}\sigma_{1}\left(a\right),\ldots ,\sqrt{\tau_{s_{1}}}\sigma_{s_{1}}\left(a\right),\\ \; & \sqrt{\tau_{1+s_{1}}}\sigma_{1+s_{1}}\left(a\right),\ldots ,\sqrt{\tau_{2s_{2}+s_{1}}}\sigma_{2s_{2}+s_{1}}\left(a\right)). \end{array}$$

Since τ=1 in *F* is totally positive, then $\sigma_{1}=\sigma$, which means that twisted embeddings are generalizations of the canonical embedding. Twisted embeddings provide a way to obtain a variety of lattices in $H≃R^{n}$ in addition to the ones obtained via canonical embedding, as a consequence of Proposition 1 [36].

**Proposition** **1** ([36])**.** *If M is a free Z-module of rank n in K (particularly, if M is the ring of integers of K or any fractional ideal of K), then $\sigma_{\tau }\left(M\right)$ is a full-rank lattice in H.*

Twisted embeddings can be extended from *K* to $K_{R}$ as follows. For any totally positive element τ∈F, the R-vector space $\sigma_{\tau }\left(K_{R}\right)$ is isomorphic to $H≃R^{n}$. If B is a Q-basis of the number field *K*, then B is an R-basis of $K_{R}$. So, for all totally positive τ∈F, $\sigma_{\tau }\left(B\right)$ is an R-basis of *H*.

Consider the natural extension of the trace function ${\mathrm{Tr}}_{K}:K\to Q$ to ${\mathrm{Tr}}_{K}:K_{R}\to R$. For any totally positive τ∈F, we can define an inner product in $K_{R}$ as

(2) $${\langle a,b\rangle }_{\tau }:={\langle \sigma_{\tau }\left(a\right),\sigma_{\tau }\left(b\right)\rangle }_{H}={\mathrm{Tr}}_{K}\left(\tau a\bar{b}\right),\;a,b\in K_{R}.$$

By considering the inner product ${\langle \cdot ,\cdot \rangle }_{\tau }$, the R-vector space $K_{R}$ is an Euclidean vector space of dimension *n* isometric to both $\left(H,{\langle \cdot ,\cdot \rangle }_{H}\right)$ and $\left(R^{n},\langle \cdot ,\cdot \rangle \right)$.

For each $a\in K_{R}$, the $\ell_{p}$-norms of *a* under the canonical embedding are simply ${∥a∥}_{p}={∥\sigma \left(a\right)∥}_{p}={\left(\sum_{i\in [n]}{\left|\sigma_{i}\left(a\right)\right|}^{p}\right)}^{1/p}$ for p<∞, and $\max_{i\in [n]}\left|\sigma_{i}\left(a\right)\right|$ for p=∞. Similarly, the $\ell_{p}$-norms induced from $C^{n}$ under twisted embeddings are defined as

$${∥a∥}_{p,\tau }:={∥\sigma_{\tau }\left(a\right)∥}_{p}={\left(\sum_{i\in [n]}{\left|\sqrt{\tau_{i}}\sigma_{i}\left(a\right)\right|}^{p}\right)}^{1/p}$$

for p<∞, and the $\ell_{\infty }$-norm is

$${∥a∥}_{\infty ,\tau }:={∥\sigma_{\tau }\left(a\right)∥}_{\infty }=\max_{i\in [n]}\left|\sqrt{\tau_{i}}\sigma_{i}\left(a\right)\right|,$$

where $\tau_{i}=\sigma_{i}\left(\tau \right)$ for a totally positive element τ∈F. Thus, any free Z-module *M* of rank *n* can be seen as a full-rank lattice directly in the Euclidean vector space ($K_{R}$,${\langle \cdot ,\cdot \rangle }_{\tau }$), although the image of $\sigma_{\tau }\left(M\right)$ is frequently considered as in $\left(H,{\langle \cdot ,\cdot \rangle }_{H}\right)$.

Using the fact that $\sigma_{\tau }\left(a\cdot b\right)=\sigma \left(a\right)⊙\sigma_{\tau }\left(b\right)=\sigma_{\tau }\left(a\right)⊙\sigma \left(b\right)$ for any $a,b\in K_{R}$, where ⊙ is the component-wise multiplication in the space *H*, it follows that

(3) $${∥a\cdot b∥}_{p,\tau }\leq {∥a∥}_{\infty }{∥b∥}_{p,\tau }{\;\mathrm{and}\;∥a\cdot b∥}_{p,\tau }\leq {∥a∥}_{p}{∥b∥}_{\infty ,\tau }.$$

Notice that, since multiplication of elements in $K_{R}$ is mapped to coordinate-wise multiplication in *H*, we have that for any element $a\in K_{R}$, the distribution of $a\cdot D_{r}$ is $D_{r^{\prime }}$, where $r_{i}^{\prime }=r_{i}\cdot \left|\sqrt{\tau_{i}}\sigma_{i}\left(a\right)\right|$ for i∈[n]. Because of the induced norms from C, which maps elements of *K* to *H*, an elliptical distribution defined in the space *H* can be seen as a distribution directly over $K_{R}$. For practical applications, sampling from an error distribution in $K_{R}$ is done by generating the error in *H* and mapping it to its corresponding element in $K_{R}$, via twisted embeddings. However, in some special cases, an error can be efficiently sampled directly in $K_{R}$ without requiring the computation of the inverse of the Vandermonde matrix with respect to $\sigma_{\tau }$ [11].

Since $K_{R}≃R^{n}$ under twisted embeddings, it follows that $K_{R}$ admits an orthonormal basis. Thus, for any Z-basis $B=\{v_{1},v_{2},\ldots ,v_{n}\}$ of the free Z-module *M* of rank *n* in *K*, the matrix ${\left[{\langle v_{i},v_{j}\rangle }_{\tau }\right]}_{n\times n}$ is a Gram matrix of the lattice *M* in ($K_{R}$,${\langle \cdot ,\cdot \rangle }_{\tau }$), which coincides with the Gram matrix of $\sigma_{\tau }\left(M\right)$ in $\left(H,{\langle \cdot ,\cdot \rangle }_{H}\right)$ with respect to the basis $\left\{\sigma_{\tau }\left(v_{1}\right),\sigma_{\tau }\left(v_{2}\right),\ldots ,\sigma_{\tau }\left(v_{n}\right)\right\}$. It should be clear that, for different totally positive elements, the lattices obtained from *M* may not be equivalent, as can be seen below.

**Example** **3.** *Let $K=Q\left(\sqrt{3}\right)=\left\{a+b\sqrt{3}\;:\;a,b\in Q\right\}$ be a totally real number field with degree two. It follows that the fixed field by the usual involution is F=K. For any totally positive element τ∈F, consider the lattice $M_{\tau }=O_{K}=Z\left[\sqrt{3}\right]$ in the inner product space $\left(K_{R},{\langle \cdot ,\cdot \rangle }_{\tau }\right)$. The set $\left\{1,\sqrt{3}\right\}$ is a Z-basis of $M_{\tau }$ and the Gram matrix of the lattice $M_{\tau }$ is given by*

(4) $$G_{\tau }=\left[\begin{array}{cc} {\mathrm{Tr}}_{K}\left(\tau \right) & {\mathrm{Tr}}_{K}\left(\tau \sqrt{3}\right)\\ {\mathrm{Tr}}_{K}\left(\tau \sqrt{3}\right) & {\mathrm{Tr}}_{K}\left(3\tau \right) \end{array}\right].$$

*For example, for τ=1 and $\tau =2+\sqrt{3}$, the Gram matrices are given by:*

(5) $$G_{1}=\left[\begin{array}{cc} 2 & 0\\ 0 & 6 \end{array}\right]\;\mathrm{and}\;G_{2+\sqrt{3}}=\left[\begin{array}{cc} 4 & 6\\ 6 & 12 \end{array}\right].$$

*Suppose that these two lattices are equivalent. Then, there exists a square matrix U with integer entries and determinant ±1, and a real number k≠0 such that $G_{2+\sqrt{3}}=k^{2}U^{t}G_{1}U$. Since the determinant of both matrices in (5) is equal to *12*, then k=±1. Now, consider U to be a matrix for which the rows are given by the vectors $\left(a,b\right)\in Z^{2}$ and $\left(c,d\right)\in Z^{2}$. So, the system of equations $G_{2+\sqrt{3}}=U^{t}G_{1}U$ has no solution $\left(a,b,c,d\right)\in Z^{4}$ because the equation $2=a^{2}+3c^{2}$, provided by the first entry, has no solution $\left(a,c\right)\in Z^{2}$. This gives a contradiction. Therefore, the lattices given by the same module $M=O_{K}$ in the two different inner product spaces $\left(K_{R},{\langle \cdot ,\cdot \rangle }_{1}\right)$ and $\left(K_{R},{\langle \cdot ,\cdot \rangle }_{2+\sqrt{3}}\right)$ are not equivalent.*

Any full-rank lattice *M* in $\left(K_{R},{\langle \cdot ,\cdot \rangle }_{\tau }\right)$ is said to be an *algebraic lattice*. If M=I is a fractional ideal in *K* and the lattice I is integral (that is, ${\langle a,b\rangle }_{\tau }\in Z$ for all a,b∈I), then I can be called an *ideal lattice* in $\left(K_{R},{\langle \cdot ,\cdot \rangle }_{\tau }\right)$. Since ${\langle a,b\rangle }_{\tau }={\mathrm{Tr}}_{K}\left(\tau a\bar{b}\right)$, an ideal I of *K* constitutes an ideal lattice in $\left(K_{R},{\langle \cdot ,\cdot \rangle }_{\tau }\right)$ if and only if $\tau I\bar{I}\subset D_{K}^{-1}$ ($=O_{K}^{\vee }$). Ideal lattices can be obtained if and only if *K* is either a totally real number field or a CM-field. In particular, ideal lattices can be obtained via cyclotomic number fields and their maximal real subfields.

Let I be a fractional ideal of *K*. It is known that $\sigma \left(I^{\vee }\right)=\bar{\sigma {\left(I\right)}^{*}}$ in *H* under the canonical embedding. However, the same does not hold for twisted embeddings in general, as can be inferred from Proposition 2.

**Proposition** **2.** *Let τ∈F be a totally positive element and let I a fractional ideal of K. Then, in the Euclidean vector space $\left(K_{R},{\langle \cdot ,\cdot \rangle }_{\tau }\right)$, it follows that:*

*(i)*  $I^{*}=\tau^{-1}{\bar{I}}^{\vee }$; and
*(ii)*  *I is an unimodular (self-dual) lattice in $\left(K_{R},{\langle \cdot ,\cdot \rangle }_{\tau }\right)$ if and only if $\tau I\bar{I}=D_{K}^{-1}$.*

**Proof.**  By definition, $a\in I^{*}$ if and only if ${\mathrm{Tr}}_{K}\left(\tau a\bar{I}\right)\subset Z$, which occurs if and only if $\tau a\in {\bar{I}}^{\vee }$, which is equivalent to $a\in \tau^{-1}{\bar{I}}^{\vee }$. This proves (i). Secondly, I is unimodular when I is integral and $I=I^{*}$. The lattice I is integral if and only if $\tau I{\bar{I}}^{-1}\subset D_{K}^{-1}$. In turn, by (i), $I=I^{*}$ if and only if $I=\tau^{-1}{\bar{I}}^{\vee }=\tau^{-1}{\bar{I}}^{-1}D_{K}^{-1}$, which is equivalent to $\tau I\bar{I}=D_{K}^{-1}$. Therefore, I is unimodular if and only if $\tau I\bar{I}=D_{K}^{-1}$.    □

### 3.2. The Twisted Ring-LWE Problem

In this section, we propose an extended version of the Ring-LWE problem, adopting twisted embeddings rather than the canonical embedding. We refer to this new class of problems as *Twisted Ring-LWE*, or simply Ring-LWE${}^{\tau }$. We also prove that solving the Twisted Ring-LWE problem is at least as hard as solving the original Ring-LWE problem [4], providing a polynomial-time reduction from Ring-LWE to Twisted Ring-LWE.

In the Ring-LWE distribution, the error *e* is randomized by a distribution ψ over the space $\left(K_{R},{\langle \cdot ,\cdot \rangle }_{\tau =1}\right)$. In this sense, an error in $K_{R}$ can be seen as the inverse image of a sample from the distribution ψ in $H≃R^{n}$ via the canonical embedding. In our general case, we consider *K* a number field with an involution, *F* its associated fixed field, τ∈F a totally positive element, and $\sigma_{\tau }$ the twisted embedding. The error *e* is randomized by a distribution ψ over $\left(K_{R},{\langle \cdot ,\cdot \rangle }_{\tau }\right)$. In the following, it is assumed q≥2 is an integer number, $R:=O_{K}$, and $I_{q}:=I/qI$ for any fractional ideal I of *K*.

**Definition** **11** (Twisted Ring-LWE distribution)**.** *For a totally positive element τ∈F, let $\psi_{\tau }$ denote an error distribution over the inner product ${\langle \cdot ,\cdot \rangle }_{\tau }$ and $s\in R_{q}^{\vee }$ (the “secret”) be an uniformly randomized element. The* Twisted Ring-LWE distribution*$A_{s,\psi_{\tau }}$ produces samples of the form*

(6) $$\left(a,\;b=a\cdot s+e\;\mathrm{mod}\;qR^{\vee }\right)\in R_{q}\times K_{R}/qR^{\vee },$$

*where a is uniformly randomized in $R_{q}$ and the error e is randomized by $\psi_{\tau }$ in $\left(K_{R},{\langle \cdot ,\cdot \rangle }_{\tau }\right)$.*

Analogously to Ring-LWE [4], which is defined in the space $K_{R}$ provided with the inner product associated to the canonical embedding, we can define both search and decision problems in the space $\left(K_{R},{\langle \cdot ,\cdot \rangle }_{\tau }\right)$ as follows. We strictly follow the search problem as defined by Lyubashevsky et al. [4] and the decision problem which was further defined by Peikert et al. [9].

**Definition** **12.** *For a positive real α>0, the family $\Psi_{\leq \alpha }^{\left(\tau \right)}$ is the set of all elliptical Gaussian distributions $D_{r}$ over $\left(K_{R},{\langle \cdot ,\cdot \rangle }_{\tau }\right)$, where each parameter $r_{i}\leq \alpha$.*

**Definition** **13** (Ring-LWE${}^{\tau }$, search)**.** *Let $\Psi^{\left(\tau \right)}$ be a family of distributions over the inner product space ($K_{R}$,${\langle \cdot ,\cdot \rangle }_{\tau }$). The search version of the Ring-LWE${}^{\tau }$ problem is defined as follows: given access to arbitrarily many independent samples from $A_{s,\psi_{\tau }}$ for some arbitrary $s\in R_{q}^{\vee }$ and $\psi_{\tau }\in \Psi^{\left(\tau \right)}$, find s.*

**Definition** **14.** *Fix an arbitrary $f\left(n\right)=\omega \left(\sqrt{\log n}\right)$. For α>0, a distribution sampled from $\Upsilon_{\alpha }^{\left(\tau \right)}$ is an elliptical Gaussian $D_{r}$ in $\left(K_{R},{\langle \cdot ,\cdot \rangle }_{\tau }\right)$, where r is sampled as follows: for $i\in [s_{1}]$, sample $x_{i}\leftarrow D_{1}$ and set $r_{i}^{2}=\alpha^{2}\left(x_{i}^{2}+f^{2}\left(n\right)\right)/2$. For $i=s_{1}+1,\ldots ,s_{1}+s_{2}$, sample $x_{i},y_{i}\leftarrow D_{1/\sqrt{2}}$ and set $r_{i}^{2}=r_{i+s}^{2}=\alpha \left(x_{i}^{2}+y_{i}^{2}+f^{2}\left(n\right)\right)/2$.*

Notice that, in Definition 14, sampling $x_{i}\leftarrow D_{1}$ for $i\in [s_{1}]$ and $x_{i},y_{i}\leftarrow D_{1/\sqrt{2}}$ for $i=s_{1}+1,\ldots ,s_{1}+s_{2}$ is done according to the Gaussian function given in Equation (1), using the norm induced by the corresponding twisted embedding.

**Definition** **15** (Ring-LWE${}^{\tau }$, average-case decision)**.** *Let $\Upsilon^{\left(\tau \right)}$ be a distribution over a family of error distributions, each in the inner product space ($K_{R}$,${\langle \cdot ,\cdot \rangle }_{\tau }$). The average-case decision version of the Ring-LWE${}^{\tau }$ problem is to distinguish, with non-negligible advantage, between arbitrarily many independent samples from $A_{s,\psi_{\tau }}$, for a random choice of $\left(s,\psi_{\tau }\right)\leftarrow U\left(R_{q}^{\vee }\right)\times \Upsilon^{\left(\tau \right)}$, and the same number of uniformly random and independent samples from $R_{q}\times K_{R}/R^{\vee }$.*

Generally speaking, the Twisted Ring-LWE distribution and both search and decision variants of Twisted Ring-LWE collapse to their original definitions in the Ring-LWE problem when τ=1.

### 3.3. Hardness of Twisted Ring-LWE

In this section we provide evidence of the hardness of the Ring-LWE${}^{\tau }$ class of problems. Firstly, we provide reductions from the Ring-LWE problem to the Ring-LWE${}^{\tau }$ problem. By doing so, the Ring-LWE${}^{\tau }$ problem is proven to be at least as hard as NP-hard lattice problems. It occurs that these are indeed self reductions, in the sense that they preserve the secret term $s\in R_{q}^{\vee }$, only distorting the error distribution over $K_{R}$.

We recall that the reduction to the search version of Ring-LWE is defined over a set of elliptical Gaussian distributions over $K_{R}$ (Definition 12).

**Theorem** **1.** *Let K be an arbitrary number field and τ∈F be totally positive. Let (s,ψ) be randomly chosen from $\left(U\left(R_{q}^{\vee }\right)\times \Psi \right)$ in $\left(K_{R},{\langle \cdot ,\cdot \rangle }_{\tau =1}\right)$. Then there is a polynomial-time reduction from* Ring-LWE*${}_{q,\psi }$ to* Ring-LWE*${}_{q,\psi_{\tau }}^{\tau }$.*

**Proof.**  We assume the existence of an oracle for Ring-LWE${}^{\tau }$ that, given a set of independent samples from $A_{s,\psi_{\tau }}$, for some arbitrary $s\in R_{q}^{\vee }$ and $\psi_{\tau }\in \Psi^{\left(\tau \right)}$, recovers the secret term *s*. Given a set of independent samples from the Ring-LWE distribution $A_{s,\psi }$, solving the search version of Ring-LWE amounts to finding the secret *s*. In order to evoke the Ring-LWE${}^{\tau }$ oracle to solve Ring-LWE, we must ensure that the error terms from the input samples follow a Gaussian distribution $\psi_{\tau }\in \Psi^{\left(\tau \right)}$. Let the input samples from $A_{s,\psi }$ be represented as

$$\left(a_{i},b_{i}=a_{i}\cdot s+e_{i}\;\mathrm{mod}\;qR^{\vee }\right)\in R_{q}\times T,$$

where $e_{i}\overset{\psi }{\leftarrow }K_{R}$. Thus, we use the fact that $e_{i}=\sigma^{-1}\left({\tilde{e}}_{i}\right)$, for some ${\tilde{e}}_{i}$ obtained from the Gaussian distribution ψ over *H*. The Ring-LWE${}^{\tau }$ samples are obtained by first computing the corresponding representatives of each pair $\left(a_{i},b_{i}\right)$ in *H* as

$$\begin{array}{cc} \left\{\left(\sigma \left(a_{i}\right),\sigma \left(b_{i}\right)\right)\right\} & =\left\{\left(\sigma \left(a_{i}\right),\sigma \left(a_{i}\right)\cdot \sigma \left(s\right)+{\tilde{e}}_{i}\right)\right\}. \end{array}$$

By applying the inverse transformation $\sigma_{\tau }^{-1}$, we obtain that

(7) $$\begin{array}{cc} \left\{\left(\sigma_{\tau }^{-1}\left(\sigma \left(a_{i}\right)\right),\sigma_{\tau }^{-1}\left(\sigma (b_{i})\right)\right)\right\} & =\left\{\left(\sigma_{\tau }^{-1}\left(\sigma \left(a_{i}\right)\right),\sigma_{\tau }^{-1}\left(\sigma \left(a_{i}\right)\right)\cdot s+\sigma_{\tau }^{-1}\left({\tilde{e}}_{i}\right)\right)\right\}. \end{array}$$

Notice that *s* was unchanged by the transformations, so it is a randomized element over $R_{q}^{\vee }$. Because $a_{i}$ was sampled according to a uniform distribution over $R_{q}$ and both σ and $\sigma_{\tau }^{-1}$ transformations are injective, $\sigma_{\tau }^{-1}\left(\sigma \left(a_{i}\right)\right)$ is also uniform in $R_{q}$. And, finally, since $e_{i}^{\prime }=\sigma_{\tau }^{-1}\left({\tilde{e}}_{i}\right)$ is randomized by $\psi_{\tau }$ in $\left(K_{R},{\langle \cdot ,\cdot \rangle }_{\tau }\right)$, the set of samples in (7) follows the distribution $A_{s,\psi^{\tau }}$. Given the set of samples (7) as input for the Ring-LWE${}^{\tau }$ solver, it finds the secret *s*. Then, mapping the solution to the Ring-LWE instance of the Ring-LWE${}^{\tau }$ solution is done by the identity transformation. Since the computation of the transformations σ and $\sigma_{\tau }^{-1}$ can be seen as vector-matrix multiplications, the reduction costs $O(n^{2})$ operations. Thus, the given reduction from Ring-LWE to Ring-LWE${}^{\tau }$ runs in polynomial time. This concludes the proof.    □

**Theorem** **2.** *Let K be an arbitrary number field and τ∈F be a totally positive element. Let (s,ψ) be randomly chosen from $\left(U\left(R_{q}^{\vee }\right)\times \Upsilon \right)$ in $\left(K_{R},{\langle \cdot ,\cdot \rangle }_{\tau =1}\right)$. There is a polynomial-time reduction from* Ring-LWE*${}_{q,\Upsilon }$ to* Ring-LWE*${}_{q,\Upsilon^{\left(\tau \right)}}^{\tau }$.*

**Proof.**  Given a set of *m* pairs of the form $\left(a_{i},b_{i}\right)\in R_{q}\times T$, each drawn either from $A_{s,\psi }$ or from a uniform distribution over $R_{q}\times T$, we prove that the (decision) Ring-LWE problem can be solved using only an oracle for (decision) Ring-LWE${}^{\tau }$ and a polynomial-time function for mapping the input instances. As in the reduction for the search variant, we apply the transformations σ and $\sigma_{\tau }^{-1}$, in this order, to each pair $\left(a_{i},b_{i}\right)\in R_{q}\times T$. As a result, those pairs drawn from $\left(U\left(R_{q}\right),U\left(T\right)\right)$ are still uniformly distributed over $R_{q}\times T$, since both σ and $\sigma_{\tau }^{-1}$ are injective maps. On the other hand, the pairs drawn from $A_{q,\psi }$ now follow the Ring-LWE${}^{\tau }$ distribution $A_{q,\psi_{\tau }}$. Thus, given an algorithm that solves (decision) Ring-LWE${}^{\tau }$, it distinguishes in two different sets the m/2 samples drawn from $A_{q,\psi_{\tau }}$ and those m/2 uniformly distributed. Since mapping Ring-LWE to Ring-LWE${}^{\tau }$ instances preserves distributions, the solution for (decision) Ring-LWE problem is done by an identity transformation. Finally, the computation of the transformations σ and $\sigma_{\tau }^{-1}$ costs $O(n^{2})$ operations; thus, the reduction runs in polynomial time. This concludes the proof.    □

### 3.4. Computing the Approximation Factors

Throughout this section, consider an arbitrary number field *K* of degree *n* with ring of integers $R=O_{K}$, and I a fractional ideal in *K*. Concerning the canonical embedding, a twisted embedding modifies the representatives of a fractional ideal I when seen as a lattice $\sigma_{\tau }\left(I\right)$ in *H*. Thus, since we use lattice measures such as the minimum distance and the successive minima in the security reductions, we analyze the effect of redefining the inner product in the Ring-LWE security reductions.

By strictly following the setting of Lyubashevsky et al. [4], we start by deriving upper bounds for the smoothing parameter concerning the $\ell_{p}$-norm under twisted embeddings. From the inequalities in (3), we are able to relate the $\ell_{p}$-norm under twisted embeddings with the infinity norm under the canonical embedding as

$${∥a∥}_{\infty }\geq \frac{{∥a∥}_{p,\tau }}{{\left(\sum_{i\in [n]}\tau_{i}^{p/2}\right)}^{\frac{1}{p}}}.$$

We can also relate $\ell_{p}$-norms under both embeddings in *H* as

$$\frac{1}{\max_{i\in [n]}\tau_{i}}\cdot {∥a∥}_{p,\tau }\leq {∥a∥}_{p}\leq \frac{1}{\min_{i\in [n]}\tau_{i}}\cdot {∥a∥}_{p,\tau }.$$

Using the above inequalities, Lemmas 1 and 2 present upper bounds for the smoothing parameter associated with twisted embeddings, which are a straightforward adaptation of Lemmas 2.7 and 3.5 from [42]. Notice that, when τ=1, these upper bounds are exactly the same as presented in [42]. Consider that $\lambda_{n}^{\left(p,\tau \right)}\left(\Lambda \right)$ and $\lambda_{1}^{\left(p,\tau \right)}\left(\Lambda \right)$ denotes the *k*-th successive minimum and the minimum distance of a lattice Λ in the $\ell_{p}$-norm, respectively, under a τ-twisted embedding.

**Lemma** **1.** *Let K be an arbitrary number field with fixed field by the involution F and τ∈F totally positive. For any p∈[2,∞], any n-dimensional lattice *Λ* in $\left(K_{R},{\langle \cdot ,\cdot \rangle }_{\tau }\right)$, and any ϵ>0,*

$$\eta_{\epsilon }\left(\Lambda \right)\leq \lambda_{n}^{\left(p,\tau \right)}\left(\Lambda \right)\cdot \frac{n^{1/2-1/p}}{\min_{i\in [n]}\tau_{i}}\cdot \sqrt{\log(2n(1+1/\epsilon ))/\pi }.$$

*In particular, for any $\omega (\sqrt{\log n})$ function, there is a negligible function ϵ(n) for which*

$$\eta_{\epsilon }\left(\Lambda \right)\leq \lambda_{n}^{\left(p,\tau \right)}\left(\Lambda \right)\cdot \frac{n^{1/2-1/p}}{\min_{i\in [n]}\tau_{i}}\cdot \omega \left(\sqrt{\log n}\right).$$

**Lemma** **2.** *Let K be an arbitrary number field with fixed field by the involution F and τ∈F totally positive. For any p∈[1,∞], any n-dimensional lattice *Λ* in $\left(K_{R},{\langle \cdot ,\cdot \rangle }_{\tau }\right)$, and any ϵ>0,*

$$\eta_{\epsilon }\left(\Lambda \right)\leq \frac{\max_{i\in [n]}\tau_{i}\cdot n^{1/p}\cdot \sqrt{\log(2n(1+1/\epsilon ))/\pi }}{\lambda_{1}^{\left(p,\tau \right)}\left(\Lambda^{*}\right)}.$$

*In particular, for any $\omega (\sqrt{\log n})$ function, there is a negligible function ϵ(n) such that*

$$\eta_{\epsilon }\left(\Lambda \right)\leq \max_{i\in [n]}\tau_{i}\cdot n^{1/p}\cdot \omega \left(\sqrt{\log n}\right)/\lambda_{1}^{\left(p,\tau \right)}\left(\Lambda^{*}\right).$$

The (search) Ring-LWE hardness consists in two reductions: (i) a worst-case to average-case reduction from DGS to Ring-LWE (Theorem 3); and (ii) a reduction from the Generalized Independent Vectors Problem (GIVP), which is a generalization of SIVP, to DGS (Lemma 3).

**Theorem** **3** ([4] (Theorem 4.1))**.** *Let K be an arbitrary number field of degree n with ring of integers $R=O_{K}$, and I a fractional ideal in K. Let α=α(n)>0, and let q=q(n)≥2 be such that $\alpha q\geq 2\cdot \omega (\sqrt{\log n})$. For some negligible ϵ=ϵ(n), there is a probabilistic polynomial-time quantum reduction from K-DGS${}_{\gamma }$ to R-LWE${}_{q,\Psi_{\leq \alpha }}$, where*

$$\gamma =\max\left\{\eta_{\epsilon }\left(I\right)\cdot \left(\sqrt{2}/\alpha \right)\cdot \omega \left(\sqrt{\log n}\right),\sqrt{2n}/\lambda_{1}\left(I^{\vee }\right)\right\}.$$

**Lemma** **3** ([3] (Lemma 3.17))**.** *For any $\epsilon =\epsilon \left(n\right)\leq \frac{1}{10}$ and any $\varphi \left(\Lambda \right)\geq \sqrt{2}\eta_{\epsilon }\left(\Lambda \right)$, there is a polynomial time reduction from GIVP${}_{2\sqrt{n}\varphi }$ to DGS${}_{\varphi }$.*

Thus, we use the inequalities for the smoothing parameter $\eta_{\epsilon }$ derived in Lemmas 1 and 2 to recompute the approximation factors in Theorem 3 and Lemma 3. We start by computing the approximated factor γ from Theorem 3. As long as $\alpha <\sqrt{\log n/n}$, it follows that the K-DGS${}_{\gamma }$ parameter is

$$\gamma =\eta_{\epsilon }\left(I\right)\cdot \left(\sqrt{2}/\alpha \right)\cdot \omega \left(\sqrt{\log n}\right)=\eta_{\epsilon }\left(I\right)\cdot \tilde{O}\left(1/\alpha \right).$$

Using the inequality $\eta_{\epsilon }\left(I\right)\leq \lambda_{n}^{\left(p,\tau \right)}\left(\Lambda \right)\cdot \frac{n^{1/2-1/p}}{\min_{i\in [n]}\tau_{i}}\cdot \omega \left(\sqrt{\log n}\right)$ from Lemma 1, we obtain that the parameter φ in Lemma 3 is

$$\varphi \leq \lambda_{n}^{\left(p,\tau \right)}\left(\Lambda \right)\cdot \frac{n^{1/2-1/p}}{\min_{i\in [n]}\tau_{i}}\cdot \omega \left(\sqrt{\log n}\right)\cdot \tilde{O}\left(1/\alpha \right).$$

Now, using the above inequality for φ, we define the upper bound for the GIVP parameter to be μ, for which

$$\mu =2\sqrt{n}\varphi \leq 2\sqrt{n}\cdot \lambda_{n}^{\left(p,\tau \right)}\left(\Lambda \right)\cdot \frac{n^{1/2-1/p}}{\min_{i\in [n]}\tau_{i}}\cdot \omega \left(\sqrt{\log n}\right)\cdot \tilde{O}\left(1/\alpha \right).$$

**Remark** **1.** *Notice that, regardless of the $\ell_{p}$-norm, $\mu =\tilde{O}\left(\sqrt{n}/\alpha \right)$. Since $\tilde{O}\left(\sqrt{n}/\alpha \right)$ is the approximation factor for the search version of the Ring-LWE problem [4] (Section 4), we conclude that the approximation factors remain unchanged with respect to the change of embeddings due to the asymptotic notation. Moreover, since the twisting factor is constant concerning the number field degree n, the approximation factors for the decision version of the Twisted Ring-LWE problem also remain unchanged.*

## 4. Applications of the Twisted Ring-LWE

In this section, we discuss how to extend to a more general class of number fields the results of Ducas and Durmus for sampling from a spherical Gaussian distribution [11], focusing on the algebraic realization of $Z^{n}$-lattices.

Durmus and Ducas proved a special case when a spherical Gaussian distribution with width *s* in the power basis corresponds to a spherical Gaussian distribution with width $s\sqrt{m^{\prime }}$ over the space *H* (Theorem 4) [11]. In order to sample directly over the cyclotomic ring $Q\left[x\right]/(\Phi_{m}\left(x\right))$, leading to the correct distribution in the embedding representation, they sample the error polynomial in the ring $Q\left[x\right]/(\Theta_{m}\left(x\right))$, where $\Theta_{m}\left(x\right)=x^{m}-1$ if *m* is odd, and $\Theta_{m}\left(x\right)=x^{\frac{m}{2}}+1$ if *m* is even. Then, the reduction modulo $\Phi_{m}$ leads to the correct distribution under the canonical embedding. This method avoids resorting to complex embeddings and the inverse of the Vandermonde matrix.

In the statement of Theorem 4, let $m^{\prime }=m$ if *m* is odd and $m^{\prime }=m/2$ if *m* is even. Also, let β represent the polynomial reduction from $Q\left[x\right]/(\Theta_{m}\left(x\right))$ to $Q\left[x\right]/(\Phi_{m}\left(x\right))$, and let the linear operator T:H→H with matrix in the canonical basis of *H* be:

(8) $$T=\frac{1}{\sqrt{2}}\left(\begin{array}{cc} {\mathrm{Id}}_{\phi (m)/2} & i{\mathrm{Id}}_{\phi (m)/2}\\ {\mathrm{Id}}_{\phi (m)/2} & -i{\mathrm{Id}}_{\phi (m)/2} \end{array}\right),\;\mathrm{with}\;i=\sqrt{-1}.$$

**Theorem** **4** ([11] (Theorem 5))**.** *Let $v\in Q\left[x\right]/(\Theta_{m}\left(x\right))$ be a random variable distributed as $\psi_{s}^{m^{\prime }}$ in the power basis. Then, the distribution of $\left(T^{-1}\circ \sigma \circ \beta \right)\left(v\right)$, seen in the canonical basis of H, is the spherical Gaussian $\psi_{s\sqrt{m^{\prime }}}^{\phi (m)}$.*

The shape of the distribution is preserved because the transformation $T^{-1}\circ \sigma$ is, in fact, a scaled-orthogonal map from the power basis of $Q\left[x\right]/(\Phi_{m}\left(x\right))$ to the space *H*, where $T^{-1}$ is Hermitian ($T^{-1}={\bar{T}}^{t}$). The proof for Theorem 4 reduces to proving that $M\in C^{\phi \left(m\right)\times m^{\prime }}$, the matrix representing the linear map γ from the power basis of $Z\left[x\right]/(\Theta_{m}\left(x\right))$ to the canonical basis of $C^{\phi (m)}$ satisfies $C=M{\bar{M}}^{t}=m^{\prime }{\mathrm{Id}}_{\phi (m)}$. The coefficients of M are given by $m_{i,j}=\sigma_{j}\left(x^{i}\right)=\zeta_{m}^{ij}$. Then, for all $i,j\in Z_{m}^{*}$, we have that

$$c_{i,j}=\sum_{k\in [m^{\prime }]}\zeta_{m}^{ik}\bar{\zeta_{m}^{jk}}=\sum_{k\in [m^{\prime }]}{\left(\zeta_{m}^{i-j}\right)}^{k}=\left\{\begin{array}{cc} m^{\prime }\; & \mathrm{if}\;i=j,\\ 0\; & \mathrm{otherwise}. \end{array}\right.$$

Thus, $E=T^{-1}M=\bar{E}$, so $EE^{t}=E{\bar{E}}^{t}=T^{-1}M{\bar{M}}^{t}T=m^{\prime }{\mathrm{Id}}_{\phi (m)}$. This last equation implies that, if a random variable $v\in Q\left[x\right]/(\Theta_{m}\left(x\right))$ has covariance matrix $s^{2}{\mathrm{Id}}_{m^{\prime }}$, then the covariance matrix of $\left(T^{-1}\circ \gamma \right)\left(v\right)$ is $s^{2}E{\mathrm{Id}}_{m^{\prime }}{\bar{E}}^{t}=s^{2}m^{\prime }{\mathrm{Id}}_{\phi (m)}$, and the distribution of $\left(T^{-1}\circ \gamma \right)\left(v\right)$ is the spherical Gaussian $\psi_{s\sqrt{m^{\prime }}}^{\phi (m)}$.

In the following, we discuss how the shape of spherical Gaussian distributions may be preserved when seen in the space *H* for special algebraic constructions under twisted embeddings. Following Ducas and Durmus’ approach, we are interested in lattices equivalent to $Z^{n}$, whose Gram matrices have the form $c{\mathrm{Id}}_{n}$ for c∈R. In this sense, the matrix mapping elements of $K_{R}$ to the space *H* is a scaled-orthogonal map [11]. It follows that any algebraic realization of the $Z^{n}$-lattice preserves the shape of an error distribution over $K_{R}$ when seen as in *H*.

In Theorem 5, we prove that fractional ideals realizing lattices equivalent to $Z^{n}$ in an orthonormal basis, which are the special case when the Gram matrix is simply ${\mathrm{Id}}_{n}$, preserve both format and standard deviation of spherical Gaussian distributions. We recall that ideal lattices can be obtained if and only if *K* is a totally real number field, or if *K* is a CM-field [36].

**Theorem** **5.** *Let K be a number field with an involution and F its associated fixed field. Consider τ∈F totally positive and $I\subset O_{K}$ a fractional ideal such that I is an ideal lattice in $\left(K_{R},{\langle \cdot ,\cdot \rangle }_{\tau }\right)$. If I is a lattice equivalent to $Z^{n}$, then both the shape and the standard deviation of a spherical Gaussian distribution in an orthonormal basis of $I\subset K_{R}$ are preserved when seen in the canonical basis of the space H (via the twisted embedding $\sigma_{\tau }$).*

**Proof.**  Let *n* be the degree of *K* and let v∈I be a random variable over the spherical Gaussian distribution with covariance matrix $s^{2}{\mathrm{Id}}_{n}$ in an orthonormal Z-basis of I, for some real number *s*. Since the twisted embedding $\sigma_{\tau }:K_{R}\to H$ is a linear transformation, the covariance matrix of $\sigma_{\tau }\left(v\right)$ in the canonical basis of *H* is $Es^{2}{\mathrm{Id}}_{n}E^{t}$, where $E=T^{-1}M$, with T as in (8) and M is the generator matrix of $\sigma_{\tau }\left(I\right)$. Since $MM^{t}=M^{t}M={\mathrm{Id}}_{n}$, and because $MM^{t}$ is the Gram matrix of the $Z^{n}$-equivalent lattice I in $\left(K_{R},{\langle \cdot ,\cdot \rangle }_{\tau }\right)$, the covariance matrix of $\sigma_{\tau }\left(v\right)$ is

$$Es^{2}{\mathrm{Id}}_{n}E^{t}=T^{-1}Ms^{2}{\mathrm{Id}}_{n}M^{t}T=s^{2}{\mathrm{Id}}_{n},$$

which proves that $\sigma_{\tau }\left(v\right)$ is randomized in the spherical Gaussian distribution over the canonical basis of *H* with the same standard deviation as *v* over $K_{R}$ in the orthonormal basis of I. This concludes the proof.    □

Examples of ideal lattices equivalent to $Z^{n}$ are those obtained from cyclotomic number fields $Q(\zeta_{2^{k}})$ [36], and their maximal real subfields [37], and the maximal real subfields $Q(\zeta_{p}+\zeta_{p}^{-1})$ for any prime p≥5 [43]. The case of the power-of-two cyclotomic number fields were previously addressed by Lyubashevsky et al. [4], and Ducas and Durmus [11]. In the following, we discuss the family of lattices equivalent to $Z^{n}$ built on $Q(\zeta_{p}+\zeta_{p}^{-1})$, for any p≥5 prime.

Let p≥5 be a prime number, n=(p−1)/2, and $\zeta =\zeta_{p}=\exp\left(-2i\pi /p\right)$. The cyclotomic construction of the $Z^{n}$-lattice (Proposition 3) is on the ring of integers of the maximal real subfield of a cyclotomic number field, denoted $Q(\zeta +\zeta^{-1})$, whose integral basis is $C=\{e_{j}=\zeta^{j}+\zeta^{-j}|1\leq j\leq n\}$.

**Proposition** **3** ([44] (Proposition 1)). *Let p≥5 be a prime number, and let $K=Q(\zeta_{p}+\zeta_{p}^{-1})$ and $\tau =\frac{1}{p}\left(1-\zeta_{p}\right)\left(1-\zeta_{p}^{-1}\right)$. Then $O_{K}$ in $\left(K_{R},{\langle \cdot ,\cdot \rangle }_{\tau }\right)$ is a lattice equivalent to $Z^{n}$ with basis $C^{\prime }=\left\{e_{1}^{\prime },\ldots ,e_{n}^{\prime }|e_{n}^{\prime }=e_{n}\;\mathrm{and}\;e_{j}^{\prime }=e_{j}+e_{j+1}^{\prime }\right\}$, where $C=\{e_{1},\ldots ,e_{n}\}$ is an integral basis of K.*

The generator matrix of the $Z^{n}$-lattice in $H=R^{n}$ (this is an equality because *K* is totally real), realized in Proposition 3, is given by

(9) $$M=DM^{\prime }U,$$

where $D=\mathrm{diag}{\left[\sqrt{\frac{\sigma_{k}\left(\tau \right)}{p}}\right]}_{n\times n}$, $M^{\prime }={\left[\sigma_{i}\left(\zeta^{j}+\zeta^{-j}\right)\right]}_{i,j\in [n]\times [n]}$ and

$$U={\left[\begin{array}{cccccc} 1 & 0 & 0 & \cdots & 0 & 0\\ 1 & 1 & 0 & \cdots & 0 & 0\\ 1 & 1 & 1 & \cdots & 0 & 0\\ \vdots & \vdots & \vdots & \ddots & \vdots & \vdots \\ 1 & 1 & 1 & \cdots & 1 & 1 \end{array}\right]}_{n\times n}.$$

As an immediate consequence of Theorem 5, in Corollary 1 we prove that the construction for the $Z^{n}$-lattice mentioned above, in fact does not change the shape of the error distribution and, more importantly, the standard deviation is the same when the distribution is seen over *H*.

**Corollary** **1.** *Let $K=Q(\zeta_{p}+\zeta_{p}^{-1})$ for p≥5 prime and let $v\in O_{K}$ be a random variable distributed as $\psi_{s}^{n}$ in the basis $C^{\prime }$. Then, the distribution of $\left(T^{-1}\circ \sigma_{\tau }\right)\left(v\right)$ for $\tau =\frac{1}{p}\left(1-\zeta_{p}\right)\left(1-\zeta_{p}^{-1}\right)$, seen in the canonical basis of H, is the spherical Gaussian $\psi_{s}^{n}$.*

**Proof.**  In the realization of the $Z^{n}$-lattice (Proposition 3), the matrix representing the linear map $\sigma_{\tau }$ from the basis $C^{\prime }$ of $O_{K}$ to the canonical basis of $R^{n}$ is given by M (9). Since $O_{K}$ is a lattice equivalent to $Z^{n}$ in the basis $C^{\prime }$, the result follows immediately from Theorem 5. This concludes the proof.    □

### 4.1. Practical Impacts on a Public-Key Cryptosystem

In this section, we use the fact that $K=Q(\zeta_{p}+\zeta_{p}^{-1})$ is a subfield of $Q(\zeta_{p})$, for *p* prime, to analyze the practical impacts of instantiating the Ring-LWE problem over the ring of integers of *K* in the compact public-key cryptosystem of Lyubashevsky, Peikert, and Regev [12] (Section 8.2).

The public-key cryptosystem presented below is parameterized by an *m*-th cyclotomic ring *R* and two coprime integers $p^{\prime }$ and *q*. The message space is defined as $R_{p^{\prime }}$ and it is required that *q* be coprime with every odd prime dividing *m*. Consider that $\psi_{\tau }$ is an error distribution over $\left(K_{R},{\langle \cdot ,\cdot \rangle }_{\tau }\right)$ and ⌊·⌉ denotes a valid discretization to (cosets) of $R^{\vee }$ or $p^{\prime }R^{\vee }$. Also, $\hat{m}=m/2$ if *m* is even, otherwise $\hat{m}=m$. Finally, for any $\bar{a}\in Z_{q}$, let $〚\bar{a}〛$ denote the unique representative $a\in \left(\bar{a}+qZ\right)\cap \left[-q/2,q/2\right)$, which is entry-wise extended to polynomials.

- Gen: choose a uniformly random $a\in R_{q}$. Choose $x\leftarrow {\left\lfloor \psi_{\tau }\right\rceil }_{R^{\vee }}$ and $e\leftarrow {\left\lfloor p^{\prime }\cdot \psi_{\tau }\right\rceil }_{p^{\prime }R^{\vee }}$. Output $\left(a,b=\hat{m}\left(a\cdot x+e\right)\;\mathrm{mod}\;qR\right)\in R_{q}\times R_{q}$ as the public key, and *x* as the secret key.
- Enc${}_{\left(a,b\right)}\left(\mu \in R_{p^{\prime }}\right)$: choose $z\leftarrow {\left\lfloor \psi_{\tau }\right\rceil }_{R^{\vee }},e^{\prime }\leftarrow {\left\lfloor p^{\prime }\cdot \psi_{\tau }\right\rceil }_{p^{\prime }R^{\vee }}$, and $e^{\prime \prime }\leftarrow {\left\lfloor p^{\prime }\cdot \psi_{\tau }\right\rceil }_{t^{-1}\mu +p^{\prime }R^{\vee }}$. Let $u=\hat{m}\left(a\cdot z+e^{\prime }\right)\;\mathrm{mod}\;qR$ and $v=z\cdot b+e^{\prime \prime }\in R_{q}^{\vee }$. Output $\left(u,v\right)\in R_{q}\times R_{q}^{\vee }$.
- Dec${}_{x}\left(u,v\right)$: compute $v-u\cdot x\;\mathrm{mod}\;qR^{\vee }$, and decode it to $d=〚v-u\cdot x〛\in R^{\vee }$. Output $\mu =t\cdot d\;\mathrm{mod}\;p^{\prime }R$.

In such an encryption scheme, the most computationally expensive operations are given by the error sampling and the discretization of the error terms, and the polynomial multiplication. As proved in Corollary 1, when *R* is the ring of integers of $Q(\zeta_{p}+\zeta_{p}^{-1})$, the sampling of error terms can be performed directly over $\left(K_{R},{\langle \cdot ,\cdot \rangle }_{\tau }\right)$ in the orthonormal basis $C^{\prime }$ while preserving the spherical format and the standard deviation with respect to the corresponding distribution in *H*. In this case, the error sampling is similar to that performed when *K* is a cyclotomic field with dimension a power of two, where the spherical format is preserved but the standard deviation increases by $m^{\prime }$. Because of that, any algorithm for one-dimensional discrete Gaussian sampling can be used in our instantiation, including those already adopted in the power-of-two cyclotomic case. The efficiency of discrete sampling when $K=Q(\zeta_{p}+\zeta_{p}^{-1})$ is emphasized by the fact that the discretization in $Z^{n}$-lattices is simply a coordinate-wise rounding to the nearest integer.

In Ring-LWE cryptosystems, arithmetic operations such as addition and multiplication are performed in the polynomial representation of the ring of integers. The ring of integers of the maximal real subfield $Q(\zeta_{p}+\zeta_{p}^{-1})$ is $Z[\zeta_{p}+\zeta_{p}^{-1}]$. Thus, associating $\zeta_{p}+\zeta_{p}^{-1}$ with indeterminate *x* yields an isomorphism between $Z[\zeta_{p}+\zeta_{p}^{-1}]$ and $Z\left[x\right]/(\Psi_{p}\left(x\right))$, where $\Psi_{p}\left(x\right)$ is the minimal polynomial of $\zeta_{p}+\zeta_{p}^{-1}$. This would require a change of basis from $C^{\prime }$, the basis used for error sampling, to the power basis $\left\{{\left(\zeta_{p}+\zeta_{p}^{-1}\right)}^{j}|0\leq j<n\right\}$. The coefficients of the defining polynomial $\Psi_{p}\left(x\right)$ vary according to the choice of *p*. Aranés and Arenas provided a closed formula for the coefficients of $\Psi_{p^{\upsilon }}\left(x\right)$ for *p* prime and υ≥1 (Theorem 7). Consider that, for strictly positives *r* and *k*, $A_{r}\left(k\right)$ are the determinants of order *k*, defined in Theorem 6. For details, we refer the reader to [45].

**Theorem** **6** ([45] (Theorem 1))**.** *For any strictly positive integers r and k, we have that*

Ar(k)=r+k−2k+r+k−3k−1,

*where nk denotes the binomial coefficient $\frac{n!}{k!(n-k)!}$.*

**Theorem** **7** ([45] (Theorem 2))**.** *The coefficients $a_{j}$ of the polynomial $\Psi_{p^{\upsilon }}\left(x\right)$ are given by the following formulae. If p is odd,*

$$a_{j}=\left\{\begin{array}{cc} 0, & \mathrm{if}\;j>m-p^{\upsilon -1};\\ \sum_{\begin{array}{c} k=1\\ k\equiv 1\;(\mathrm{mod}\;2) \end{array}}^{\left[\frac{m-j}{p^{\upsilon -1}}\right]}{\left(-1\right)}^{(m-j-kp^{\upsilon -1})/2}A_{j+2}\left(\frac{m-j-kp^{\upsilon -1}}{2}\right), & \mathrm{if}\;m+j\equiv 1\;(\mathrm{mod}\;2);\\ {\left(-1\right)}^{\frac{m-j}{2}}\sum_{k=0}^{\left[\frac{m-j}{2p^{\upsilon -1}}\right]}{\left(-1\right)}^{k}A_{j+2}\left(\frac{m-j}{2}-kp^{\upsilon -1}\right), & \mathrm{if}\;m+j\equiv 0\;(\mathrm{mod}\;2); \end{array}\right.$$

*and in the case p=2, υ≥3:*

$$a_{j}=\left\{\begin{array}{cc} {\left(-1\right)}^{\frac{m-j}{2}}A_{j+2}\left(\frac{m-j}{2}\right), & if\;j\;is\;even;\\ 0, & otherwise. \end{array}\right.$$

Notice that, in our case, υ=1; thus, all coefficients are always non-zero. For example, when p=31, we have that n=15 and the defining polynomial $\Psi_{p}\left(x\right)$ is

$$\begin{array}{cc} \Psi_{31}\left(x\right) & =x^{15}+x^{14}-14x^{13}-13x^{12}+78x^{11}+66x^{10}-220x^{9}-165x^{8}\\ \; & +330x^{7}+210x^{6}-252x^{5}-126x^{4}+84x^{3}+28x^{2}-8x-1, \end{array}$$

which is very dense and the coefficients are not restricted to the set {0,1}. However, depending on the choice of value for the coefficient’s modulus *q*, the defining polynomial may have a complete factorization modulo *q*, which allows algorithms based on the Chinese Remainder Theorem (CRT) for efficient polynomial multiplication. For example, for p=31 and q=61, the defining polynomial factors in 15 distinct degree-one polynomials as follows:

(10) $$\begin{array}{c} \Psi_{31}\left(x\right)\;\mathrm{mod}\;61=\left(x+5\right)\left(x+6\right)\left(x+15\right)\left(x+16\right)\left(x+21\right)\left(x+22\right)\left(x+24\right)\left(x+27\right)\\ (x+29)(x+36)(x+38)(x+41)(x+48)(x+49)(x+51). \end{array}$$

Thus, $f\left(x\right)=\Psi_{31}\left(x\right)$ can be factored as $f\left(x\right)=\prod_{i\in [k]}f_{i}\left(x\right)\;\left(\mathrm{mod}\;q\right)$, where $f_{i}\left(x\right)$ are polynomials of small degree. The multiplication a·b modulo f(x) is done by computing $a_{i}=a\;\mathrm{mod}\;f_{i}\left(x\right)$ and $b_{i}=b\;\mathrm{mod}\;f_{i}\left(x\right)$, for i∈[k], computing the component-wise multiplication $\left(a_{i}b_{i}\right)$ and, finally, using the inverse operation to obtain the polynomial *c* such that $c\;\mathrm{mod}\;f_{i}\left(x\right)=a_{i}b_{i}\;\mathrm{mod}\;f_{i}\left(x\right)$, as discussed by Lyubashevsky and Seiler [27]. Although the asymptotic cost of an algorithm based on this technique is O(nlogn), the hidden constants may be large due to the increased number of reductions modulo *q* in comparison with CRT-based algorithms for power-of-two cyclotomic number fields [27,46]. Another important aspect of the defining polynomial is captured by the *expansion factor*, a property introduced by Lyubashevsky and Micciancio [47]. The expansion factor of a polynomial *f* is

$$\mathrm{EF}\left(f,k\right)=\max_{g\in Z[x],\deg(g)\leq k(\deg(f)-1)}{∥g∥}_{f}/{∥g∥}_{\infty },$$

where ${∥g∥}_{f}$ is the norm of the polynomial *g* after reduction modulo *f*. By computing the expansion factor of $\Psi_{p}\left(x\right)$, we can measure the increase in magnitude of the maximum coefficient of ${∥g∥}_{\Psi_{p}\left(x\right)}$. Also, the expansion factor helps us in choosing a value for *q* such that the coefficients do not wrap around after arithmetic operations, avoiding the occurrence of decryption errors.

In order to analyze the expansion factor of $\Psi_{p}\left(x\right)$, we compare it with $x^{n}+1$, the defining polynomial of cyclotomic polynomial rings with dimension a power of two, which is widely adopted in practical applications. For that, we recall Lemma 4, which defines an upper bound for the magnitude of the coefficients of a polynomial g∈Z[x] after a reduction modulo *f*.

**Lemma** **4.** *If g is a polynomial in Z[x] and f is a monic polynomial in Z[x] such that deg(g)≥deg(f), then ${∥g∥}_{f}\leq {∥g∥}_{\infty }{\left({2∥f∥}_{\infty }\right)}^{\deg(g)-\deg(f)+1}$.*

For the case $f\left(x\right)=\Psi_{p}\left(x\right)$, it is sufficient to analyze the value of ${∥f∥}_{\infty }$. Firstly, for $f\left(x\right)=x^{n}+1$, we have that ${∥f∥}_{\infty }=1$. On the other hand, when $f\left(x\right)=\Psi_{p}\left(x\right)$, ${∥f∥}_{\infty }$ assumes the maximum value of $a_{j}$ according to Theorem 7. For example, for p=31, ${∥f∥}_{\infty }=330$, leading to an exponential growth of coefficients, which is roughly $330^{\deg(g)-\deg(f)+1}$ times bigger with respect to the case when $f\left(x\right)=x^{16}+1$. Such growth of coefficients require an increased value for the choice of the modulus *q* in order to avoid the coefficients to wrap around after polynomial operations. This also leads to an increase in the length of system parameters and memory/bandwidth requirement for transmission of public parameters.

In the positive direction, since the dimension of *K* does not increase as a power-of-two, one may want to find a ring instantiation that closely achieves a target security level. For example, to obtain a ring dimension between 700 and 800, the required for achieving 128-bit security [27], possible choices for the value of *p* ranges from the 223-th to the 252-th prime number, comprehending 29 possible choices.

In a nutshell, we have discussed some practical impacts of instantiating the Twisted Ring-LWE problem when *K* is the maximal real subfield of a cyclotomic number field, whose dimension is n=(p−1)/2 for any prime p≥5. The increased cost in arithmetic operations is inherent to this particular instantiation and field representation, but the same cannot be said about all algebraic constructions which lead to lattices equivalent to $Z^{n}$. This is reinforced by the fact that the ring of integers of power-of-two cyclotomic number fields also leads to lattices equivalent to $Z^{n}$ and, yet, it allows for very efficient algorithms for arithmetic operations in the power basis representation. Thus, in Section 5, we briefly discuss on an alternative field representation when *K* is the maximal real subfield of a cyclotomic number field. Moreover, we present future research possibilities related to the Twisted Ring-LWE problem.

## 5. Discussion

In this paper, we introduce an extension to the Ring-LWE class of problems, namely The Twisted Ring-LWE Problem [4,9]. The Ring-LWE problem uses the canonical embedding to map some underlying ring to a lattice in $R^{n}$. By doing so, we can define geometric norms and error distributions on the tensor field $K_{R}$, which is isomorphic to $R^{n}$. The Twisted Ring-LWE problem is obtained by adopting twisted embeddings [36] rather than the canonical embedding, which is a specialization of twisted embeddings. We prove that the Twisted Ring-LWE Problem is as secure as the original Ring-LWE Problem by providing a security reduction from both variants of Ring-LWE to their twisted forms.

As a result, we broaden the scope of number of algebraic lattices that can be used for lattice-based cryptosystems, including those algebraic constructions of lattices that allow additional factors on the embedding coordinates. This type of construction has been useful in coding theory, since they allow the construction of algebraic lattices with improved properties for Rayleigh fading channels, providing high density, maximum diversity, and great minimum product distance [33,34,35]. Notice that these constructions cannot be obtained via canonical embedding. We took as an example the construction of rotated $Z^{n}$-lattices. We prove that we can perform efficient and secure sampling from spherical Gaussian distributions in $K_{R}$, if the parameter ring leads to a rotated $Z^{n}$-lattice in the space *H* via twisted embeddings. This generalizes the results of Ducas and Durmus in Theorem 5 [11] and the power-of-two cyclotomic case.

An example of a construction of the $Z^{n}$-lattice via twisted embeddings is from maximal real subfields of both power-of-two and *p*-th cyclotomic number fields. We analyze instantiating the Ring-LWE problem using maximal real subfields of *p*-th cyclotomic number fields in a public-key encryption scheme [12]. By doing so, we can instantiate the Ring-LWE problem in a dimension close to 700 to achieve 128-bit security [25] and provide variability of security assumptions, avoiding the use of the widely adopted power-of-two cyclotomic number field. However, representing the field elements as residue polynomials modulo the defining polynomial is of limited interest, since the coefficients’ modulus may become very large to avoid the occurrence of decryption errors. This occurs because the expansion factor of the defining polynomial of maximal real subfields of *p*-th cyclotomic number fields grows exponentially.

### Future Work

Lyubashevsky, Peikert, and Regev [12] suggested representing the field elements as coefficient vectors in an integral basis apart from the power basis. By taking the underlying ring as the ring of integers of the maximal real subfield of a cyclotomic number field on an orthonormal basis, we can perform efficient Gaussian sampling with hardness guarantee, as discussed in Section 4. Moreover, we can perform efficient ring arithmetic by taking the ring representatives under the twisted embedding, in which both addition and multiplication are taken component-wise. Although the change of representation may need floating-point arithmetic, one may explore lattice basis symmetries to accelerate the computation of the twisted embedding or find a basis more suitable for arithmetic operations. In addition to that, all algorithmic tasks can be performed directly in the space *H*, without resorting to change of representation from $K_{R}$. We leave as future work a full analysis and the software implementation of the instantiation of the Twisted Ring-LWE Problem in a cryptosystem adopting the coefficient vector representation.

We also leave as future work detailing how to connect Twisted Ring-LWE instantiations over different number fields, if the ring of integers of both number fields leads to equivalent lattices under twisted embeddings. By doing so, we can connect an instance on a power-of-two cyclotomic number field to an instance of a maximal real subfield as both rings of integers lead to a construction of the $Z^{n}$-lattice. This may lead to a response to the open question left by Peikert, Regev, and Stephens-Davidowitz [9]. As a consequence, we may be able to explore algebraic properties inherent to maximal real subfields helping to assert the concrete hardness of power-of-two cyclotomic number fields.
